# Deep-Learning Model for Influenza Prediction From Multisource Heterogeneous Data in a Megacity: Model Development and Evaluation

**DOI:** 10.2196/44238

**Published:** 2023-02-13

**Authors:** Liuyang Yang, Gang Li, Jin Yang, Ting Zhang, Jing Du, Tian Liu, Xingxing Zhang, Xuan Han, Wei Li, Libing Ma, Luzhao Feng, Weizhong Yang

**Affiliations:** 1 Department of Management Science and Information System Faculty of Management and Economics Kunming University of Science and Technology Kunming China; 2 School of Population Medicine and Public Health Chinese Academy of Medical Sciences & Peking Union Medical College Beijing China; 3 Beijing Centre for Disease Prevention and Control Beijing China; 4 Jingzhou Center for Disease Control and Prevention Jingzhou China; 5 The First People’s Hospital of Yunnan Province The Affiliated Hospital of Kunming University of Science and Technology Kunming China; 6 Department of Respiratory and Critical Care Medicine Affiliated Hospital of Guilin Medical University Guilin China

**Keywords:** influenza, ILI, multisource heterogeneous data, deep learning, MAL model, megacity

## Abstract

**Background:**

In megacities, there is an urgent need to establish more sensitive forecasting and early warning methods for acute respiratory infectious diseases. Existing prediction and early warning models for influenza and other acute respiratory infectious diseases have limitations and therefore there is room for improvement.

**Objective:**

The aim of this study was to explore a new and better-performing deep-learning model to predict influenza trends from multisource heterogeneous data in a megacity.

**Methods:**

We collected multisource heterogeneous data from the 26th week of 2012 to the 25th week of 2019, including influenza-like illness (ILI) cases and virological surveillance, data of climate and demography, and search engines data. To avoid collinearity, we selected the best predictor according to the weight and correlation of each factor. We established a new multiattention-long short-term memory (LSTM) deep-learning model (MAL model), which was used to predict the percentage of ILI (ILI%) cases and the product of ILI% and the influenza-positive rate (ILI%×positive%), respectively. We also combined the data in different forms and added several machine-learning and deep-learning models commonly used in the past to predict influenza trends for comparison. The *R^2^* value, explained variance scores, mean absolute error, and mean square error were used to evaluate the quality of the models.

**Results:**

The highest correlation coefficients were found for the Baidu search data for ILI% and for air quality for ILI%×positive%. We first used the MAL model to calculate the ILI%, and then combined ILI% with climate, demographic, and Baidu data in different forms. The ILI%+climate+demography+Baidu model had the best prediction effect, with the explained variance score reaching 0.78, *R^2^* reaching 0.76, mean absolute error of 0.08, and mean squared error of 0.01. Similarly, we used the MAL model to calculate the ILI%×positive% and combined this prediction with different data forms. The ILI%×positive%+climate+demography+Baidu model had the best prediction effect, with an explained variance score reaching 0.74, *R^2^* reaching 0.70, mean absolute error of 0.02, and mean squared error of 0.02. Comparisons with random forest, extreme gradient boosting, LSTM, and gated current unit models showed that the MAL model had the best prediction effect.

**Conclusions:**

The newly established MAL model outperformed existing models. Natural factors and search engine query data were more helpful in forecasting ILI patterns in megacities. With more timely and effective prediction of influenza and other respiratory infectious diseases and the epidemic intensity, early and better preparedness can be achieved to reduce the health damage to the population.

## Introduction

Acute respiratory infectious diseases have long represented a threat to human health. Every large-scale outbreak of acute infectious diseases will have a significant impact on human life, health, and social development [1]. On March 11, 2020, the World Health Organization (WHO) officially declared COVID-19 as a global pandemic. To date, there have been 613,410,796 confirmed COVID-19 cases and 6,518,749 deaths worldwide [2]. In addition, an influenza pandemic may occur every 10-50 years [3]. Influenza viruses readily mutate and spread quickly. Influenza epidemics, outbreaks, and even pandemics have always threatened human health and socioeconomic development. The Spanish influenza (H1N1) outbreak in 1918 caused approximately 50 million deaths worldwide, the Asian influenza (H2N2) outbreak in 1957 caused approximately 1-2 million deaths, Hong Kong influenza (H3N2) in 1968 caused approximately 1 million deaths, and influenza A (H1N1) in 2009 caused approximately 200,000 deaths [4-7].

Owing to the constant variation in viruses and increased global connectivity, new respiratory infectious diseases have caused a heavy disease burden and economic losses to humanity. Especially in megacities, with the growth of the population and the expansion of urbanization, the contradiction between humans and animals has become increasingly significant. In addition, aging issues in megacities are of concern, with older individuals being more likely to become infected with serious acute respiratory infectious diseases. The rapid urbanization process in megacities leads to population aggregation and residential congestion. Moreover, air pollution, climate warming, and other issues in megacities have created new opportunities for the emergence of infectious diseases [8]. Therefore, in megacities, there is an urgent need to establish more sensitive forecasting and early warning methods for acute respiratory infectious diseases to monitor their epidemic trends and minimize losses.

Seasonal influenza has long been one of the major problems of public health [9]. The spread and prevalence of influenza are affected by many factors, including climate, population mobility, population immunity, and social economy. In past research, multisource data have been used for improved modeling of disease outbreaks. Ginsberg et al [10] proposed using Google search engine data to estimate the trend of influenza outbreaks, finding a high correlation between the disease search trend and the actual number of influenza infections. Subsequently, researchers began using similar data sources, including Yahoo, Wikipedia, Baidu search, and other platforms, to establish prediction models for influenza and other acute respiratory infectious diseases [11]. Some studies also found that environmental factors such as absolute humidity, temperature, and sunlight were helpful for the prediction of influenza [12,13]. China has established a national influenza surveillance system, which plays an important role in influenza surveillance. However, the national influenza surveillance system mainly analyzes confirmed influenza cases, excluding pharmacy, meteorological, and other data that may affect influenza. Therefore, the current system may have a lag in the detection of an influenza epidemic.

Surveillance approaches of influenza usually include time-series analysis, time-space analysis, infectious disease dynamics, and machine-learning and deep-learning methods [14]. Different approaches have their own advantages and disadvantages. For example, the time-series method is effective in the short-term prediction of an influenza trend, but may not be suitable for the long-term prediction of influenza [15]. The dynamic model of infectious disease can better predict the trend of an influenza epidemic from the aspect of the transmission mechanism; however, the process of model establishment is complex, and population mobility and other factors need to be considered [16]. Machine learning can process a large amount of data in a short time, which is a fast and accurate approach. Cheng et al [17] used machine-learning methods (autoregressive integrated moving average, random forest, support vector regression, and extreme gradient boosting [XGBoost]) to accurately predict the trends of influenza-like illness (ILI) in Taiwan. Choo et al [18] established a more sensitive influenza screening model by using the gated current unit (GRU) model. Jang et al [19] used news data to predict influenza, and the results showed that the long short-term memory (LSTM) model could accurately predict influenza. However, most traditional machine-learning methods may not be equipped to use multisource heterogeneous data to effectively complete prediction tasks. Therefore, although the modeling process of deep learning is also complex, it may be able to solve the problem of incorporating multisource heterogeneous data.

In this study, we integrated heterogeneous data from different sources such as ILI case data from hospitals, climate data, search engine data, and social economy data, because data from different sources may capture different influenza incidence signals in the population to varying degrees. The aim was to establish a new and better-performing deep-learning model to predict influenza trends from multisource heterogeneous data in a megacity.

## Methods

### Study Design

In this study, we chose Beijing as the research object to represent a megacity, because Beijing is the capital of the People’s Republic of China, a central national city. By the end of 2021, Beijing had more than 20 million permanent residents. Furthermore, the center of Beijing is located at a longitude of 116°20 east and a latitude of 39°56 north. Influenza is more prevalent in winter with significant peaks in this season. We first collected multisource heterogeneous data from the 26th week of 2012 to the 25th week of 2019, including ILI cases, data of virological surveillance, climate and demography data, and data from search engines. We then aligned these multisource heterogeneous data with time as the label. Simultaneously, since we observed that the absolute values of some dimensional data were not on the same order of magnitude, we normalized all data to be between 0 and 1 for further analysis and training. Because some variables may be collinear or strongly correlated, we performed correlation analysis and weight analysis to select the best data set before using the data for modeling. Finally, we performed deep-learning modeling for the filtered data of the percentage of ILI cases (ILI%) and the product of ILI% and the influenza positive rate (ILI%×positive%). We used data from the 26th week of 2012 to the 45th week at the end of 2018 to train the model, predicted the data from the 46th week of 2018 to the 25th week of 2019, and compared the obtained data with the actual data to observe the model’s fitting effect. The specific modeling process is described in detail in the Procedure section below.

### Data Collection

#### ILI Cases

The Beijing Municipal Influenza Monitoring System conducts case screening for patients in the fever clinic, internal medicine clinic, internal medicine emergency department, children’s internal medicine clinic, and children’s internal medicine emergency department of sentinel hospitals; registers those who meet the definition of influenza-like cases; and summarizes the number of influenza-like cases in each age group (0-4, 5-14, 15-24, 25-59, and ≥60 years) every day. An ILI case is defined as a patient with fever (axillary temperature≥38 ℃), cough, or sore throat. In this study, the monitoring data of ILI cases reported by more than 140 medical institutions above the third level in the Beijing Medical Institutions Infectious Disease Monitoring and Early Warning System were used for analysis. We collected ILI data from the 26th week of 2012 to the 25th week of 2019. The ILI% was calculated as the proportion of ILI case reports relative to the total number of outpatient and emergency visits in the same period.

#### Virological Surveillance

The national influenza surveillance network in China is led by the Chinese Center for Disease Control and Prevention (CDC), with 554 sentinel hospitals and 407 network laboratories. The network laboratory conducts pathogenic monitoring of influenza virus on the respiratory specimens of ILI cases and monitors the activity level and variation of influenza virus. We also obtained influenza virological surveillance data from the 26th week of 2012 to the 25th week of 2019. The average weekly positive influenza test rate was obtained by dividing the number of influenza-positive samples by the total number of samples tested during the week. We used ILI%×positive% to express the intensity of influenza activity during the study period.

#### Climate and Demography

Climate data were obtained from the China Meteorological Data Sharing Service System [20]. We collected meteorological data from Beijing from the 26th week of 2012 to the 25th week of 2019. The data comprised the mean temperature per week (Tmean), maximum temperature per week (Tmax), minimum temperature per week (Tmin), mean relative humidity (RH) per week, mean air pressure per week (Apmean), mean wind speed per week, mean precipitation per week, and mean sunlight hours per week. The absolute humidity (AH) calculation formula was as follows: AH={6.112×e^[(17.67×T)/(T±243.5)]^×RH×2.1674}/(273.15+T) [21,22]. We defined the weekly temperature difference as Tmax–Tmin. Air quality data were obtained from the Beijing Municipal Environmental Monitoring Center [23]. We also collected the mean weekly air quality data for Beijing from the 26th week of 2012 to the 25th week of 2019. Air quality was divided into six grades according to the air quality index (AQI): Grade I (excellent, AQI=0-50), Grade II (good, AQI=51-100), Grade III (slight pollution, AQI=101-150), Grade IV (moderate pollution, AQI=151-200), Grade V (severe pollution, AQI=201-300), and Grade VI (severe pollution, AQI>300). We collected the gross domestic product (GDP) data of Beijing from 2012 to 2019 and information on statutory holidays from the 26th week of 2012 to the 25th week of 2019 [24]. According to the Chinese lunar calendar, we set a holiday week at 1 and a week without a holiday at 0.

#### Search Engine

The Baidu search engine is one of the most widely used search engines in China. The Baidu search index represents the keyword search trend of many Baidu internet users, which can be used to understand internet users’ concerns and monitor public opinion trends [25]. We selected “influenza” as the keyword and the time range from the 26th week of 2012 to the 25th week of 2019. The search data were based on personal computer and personal mobile phone data.

### Procedure

#### Normalization, Correlation, and Weight Analysis

We normalized all data to fit in the range of 0-1. Because some dimensional data may have collinearity or strong correlations, to avoid the impact of collinearity on the model, we performed correlation analysis and weight analysis on all variables to select the best data set for training. The Pearson correlation coefficient (*r*) was used to measure the linear correlation in which a greater absolute value of the correlation coefficient indicates a stronger correlation; that is, a correlation coefficient closer to 1 or –1 indicates a stronger correlation and a correlation coefficient closer to 0 indicates a weaker correlation. We calculated the Pearson correlation coefficient between each variable to observe the correlations between the variables. As a traditional machine-learning model, the random forest model can score the importance of different variables and dimensional characteristics, and further evaluates the contribution of each independent variable to the dependent variable results. We constructed a random forest model to explore the influence weight of independent variables on ILI% and ILI%×positive%.

#### Model Construction

In this study, we aimed to fully explore the inherent characteristics of multisource heterogeneous data and establish the mapping from characteristics to results through building and training models to effectively predict ILI% and ILI%×positive%.

**Figure 1:**
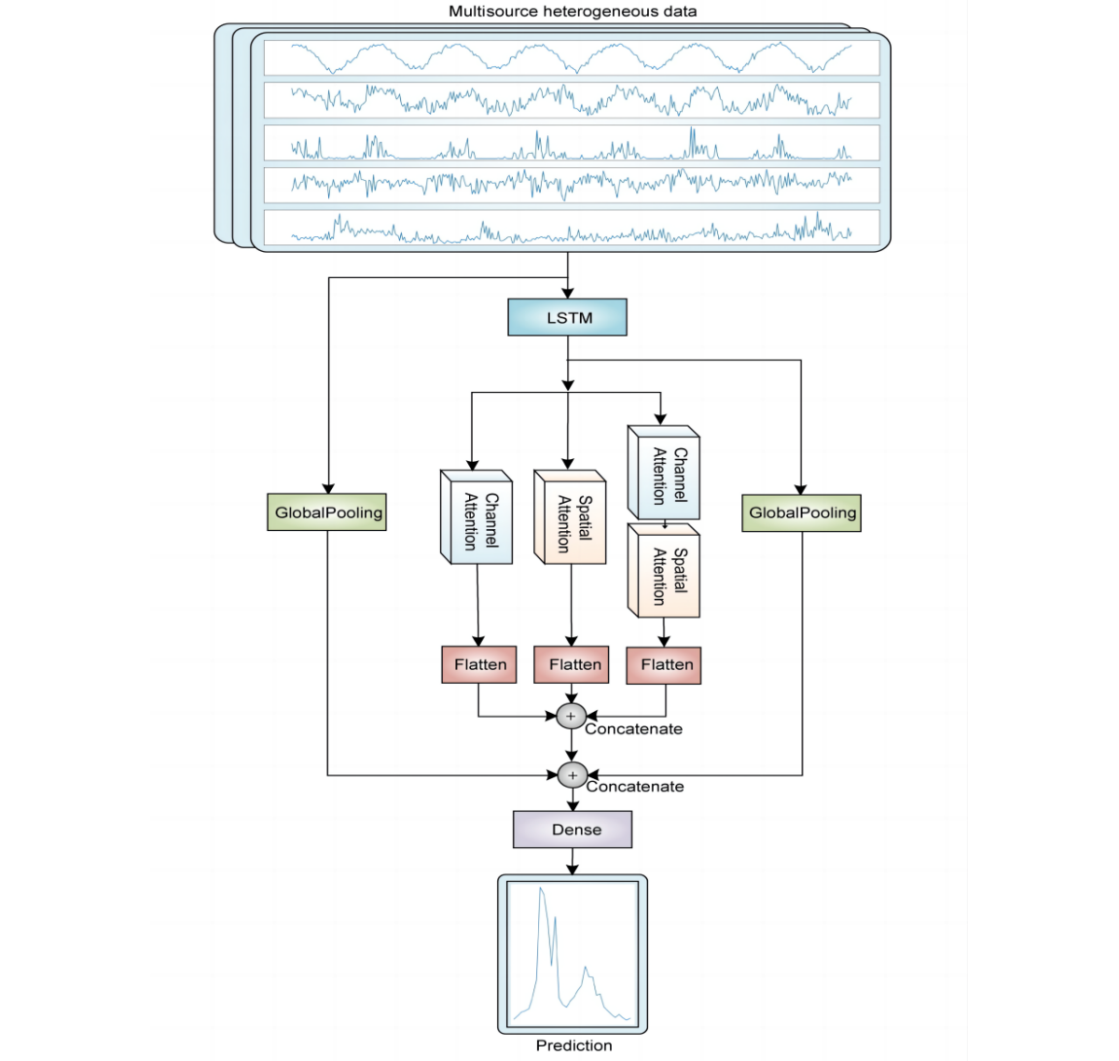
Structure of the multiattention deep-learning model (MAL) based on long short-term memory (LSTM). Schematic diagram representing multisource heterogeneous data input to the MAL model, including climate, Baidu searrch, demography, and other data. The curves of five independent variables are displayed: maximum temperature per week (Tmax), mean relative humidity per week (Hmean), mean precipitation per week, the mean temperature difference per week (dT; Tmax–Tmin), and air quality index (AQI).

Based on LSTM, we innovatively built a new multiattention deep-learning model (MAL) (Figure 1). We first set up an LSTM layer. Subsequently, three different attention modules were connected in parallel: Channel Attention, Spatial Attention, and the concatenation of Channel Attention and Spatial Attention. Different attention modules not only focuses the model on key nodes in the time series but also focuses the model on features with high weight, which can promote the improvement of model performance. We then added the flatten layers to fully obtain the information after passing the different attention modules and concatenating the three branches. To prevent the gradient from disappearing, the second concatenate was conducted after the first concatenate, with the two branches passing through the global pooling layer. Finally, a full connection was made through the dense layer, and the predicted values of ILI% and ILI%×positive% were obtained as output.

We used the data from the 26th week of 2012 to the 45th week at the end of 2018 to conduct the training model, predicted the data from the 46th week of 2018 to the 25th week of 2019, and finally compared the predicted data with the actual data to observe the model fitting effect. The following parameters were adopted for model evaluation: *R^2^*, explained variance score, mean absolute error, and mean squared error. An *R^2^* and explained variance score close to 1 indicate a good prediction effect of the model. A mean absolute error and mean squared error close to 0 further indicate the good prediction effect of the model.

### Model Comparison

To evaluate the scientific validity and robustness of the selected data set and model constructed, we compared the performance of the MAL models built with the data from ILI%+climate, ILI%+demography, ILI%+Baidu, ILI%+climate+Baidu, ILI%+climate+demography, ILI%+demography+Baidu, and ILI%+climate+demography+Baidu to verify whether the prediction effect was improved after data from different sources were fused into a model. Similarly, we compared the performance of the MAL models for the data from ILI%×positive%+climate, ILI%×positive%+demography, ILI%×positive%+Baidu, ILI%×positive%+climate+Baidu, ILI%×positive%+climate+demography, ILI%×positive%+demography+Baidu, and ILI%×positive%+climate+demography+Baidu.

Several common models such as random forest, XGBoost, GRU, and LSTM were also used for comparison with our MAL model. Random forest refers to a classifier that uses multiple trees to train and predict samples. For many data forms, random forest can produce a classifier with high accuracy. XGBoost is an integrated machine-learning algorithm based on a decision tree, which is suitable for classification, regression, sorting, and other problems. This method is fast and effective, can handle large-scale data, and supports user-defined loss functions. GRU is a gating mechanism in recurrent neural networks and can be applied to short-term or long-term prediction. LSTM is a time-recurrent neural network of a deep-learning model that can effectively solve the problem of gradient explosion or disappearance of a simple circular neural network, and is suitable for processing and predicting notable events with long intervals and delays in time series.

### Statistical Analysis

We performed all statistical analyses with Python (version 3.6.0) and Tensorflow (version 2.0.0) software.

### Ethics Considerations

This study was approved by the Ethics Committee of the Chinese Academy of Medical Sciences & Peking Union Medical College, Beijing, China (review number CAMS&PUMC-IEC-2021-032).

## Results

### Description of Data From Various Sources

We calculated the weekly average of data collected from various sources at different times. The average weekly number of ILI cases was the highest from the 26th week in 2018 to the 25th week in 2019, with 21,142 cases. We also calculated other variables as shown in Table 1.

**Figure 2:**
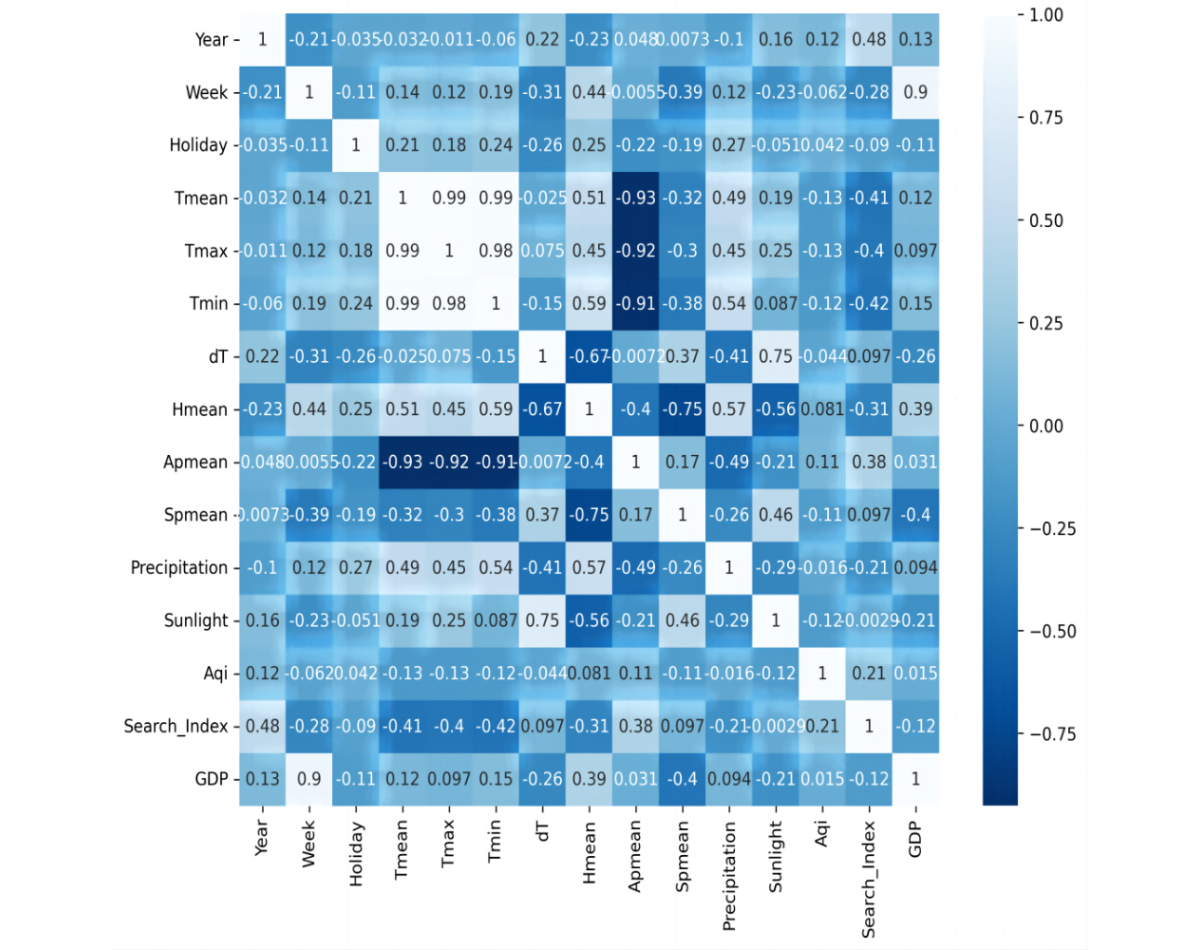
Pearson correlation analysis of different variables. Apmean: mean air pressure per week; Aqi: air quality index; dT: the weekly temperature difference; GDP: gross domestic product; Hmean: mean relative humidity per week; Spmean: mean wind speed per week; Tmax: maximum temperature per week; Tmean: mean temperature per week; Tmin: minimum temperature per week.

We built random forest models to explore the impact weight of different source variables on ILI% and ILI%×positive%. We found that Baidu’s “influenza” search index had the highest correlation with ILI% and AQI had the highest impact weight with ILI%×positive% (Table 2). To avoid collinearity between variables, we performed a correlation analysis of different variables. Tmean, Tmax, Tmin, and Apmean were highly correlated (absolute value of *r*>0.9; Figure 2). Therefore, we used Apmean with the highest weight in the modeling process for predicting ILI% and removed Tmean, Tmax, and Tmin, and used Tmean with the highest weight in the modeling process for predicting ILI%×positive% and removed Tmax, Tmin, and Apmean.

**Table 1:** Average value of different source variables for different time periods.^a^

Variables	2012-2013	2013-2014	2014-2015	2015-2016	2016-2017	2017-2018	2018-2019
ILI^b^ cases, n	13,748	12,300	12,351	13,887	14,318	19,100	21,142
Influenza-positive cases, n	26.00	37.00	47.00	39.00	30.00	45.00	45.00
Maximum temperature (℃)	16.32	18.55	18.42	18.20	18.64	17.97	18.35
Minimum temperature (℃)	5.45	6.93	6.58	6.85	7.06	6.32	6.17
Temperature difference (℃)	10.87	11.62	11.84	11.35	11.58	11.65	12.17
Mean temperature (℃)	10.53	12.29	12.12	12.22	12.46	11.77	11.90
Relative humidity (g/m^3^)	58.48	50.05	50.83	54.94	55.47	53.73	51.30
Atmosphere pressure (hPa)	993.18	992.97	993.46	993.75	993.77	993.48	994.01
Wind speed (m/s)	1.85	90.17	1.76	1.77	1.66	1.69	1.70
Precipitation (cm)	1.67	1.66	1.22	1.54	1.56	1.38	1.27
Sunlight (weeks)	6.51	6.67	6.63	6.52	6.67	6.97	7.02
AQI^c^	70.19	64.42	70.19	100.38	159.13	108.65	115.38
Baidu search index for “influenza”	117.00	205.00	218.00	215.00	284.00	428.00	443.00

^a^In each time period, data were collected from the 26th week of the first year to the 25th week of the subsequent year.

^b^ILI: influenza-like illness.

^c^AQI: air quality index.

**Table 2:** Impact weight of variables from different data sources on the proportion of influenza-like illness cases (ILI%) and the product of influenza-like illness cases and the influenza positive rate (ILI%×positive%).

Variables	Weight	
	ILI	ILI×positive
Search index	0.085	0.081
Weekly temperature difference	0.083	0.072
Sunlight	0.081	0.066
Mean air pressure per week	0.077	0.074
Mean relative humidity per week	0.076	0.067
Mean wind speed per week	0.074	0.069
Mean temperature per week	0.073	0.083
Air quality index	0.072	0.088
Minimum temperature per week	0.070	0.080
Maximum temperature per week	0.069	0.073
Week	0.066	0.067
Precipitation	0.060	0.052
Gross domestic product	0.057	0.061
Year	0.046	0.057
Holiday	0.011	0.010

### MAL of ILI% and Other Source Data

**Figure 3:**
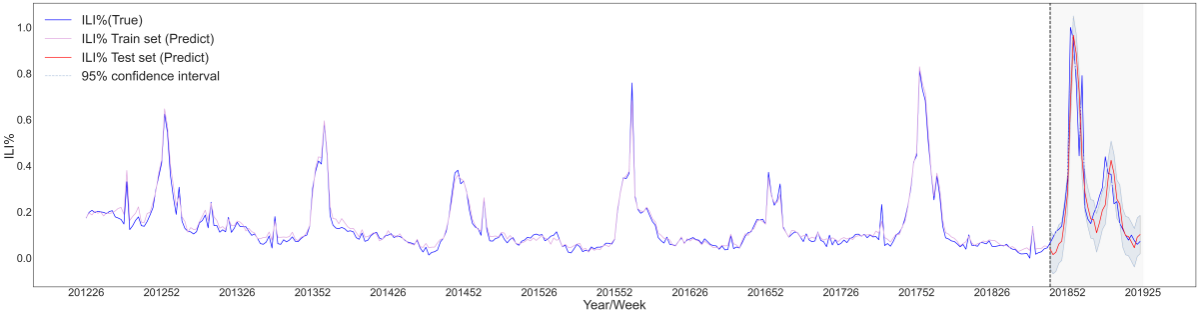
Prediction diagram for the ILI%+climate+demography+Baidu multiattention long short-term memory (MAL) deep-learning model. ILI%: percentage of influenza-like illness cases.

We first used the MAL to model ILI% and then combined and modeled ILI% with climate, demography, and Baidu data in different forms (ILI%+climate, ILI%+demography, ILI%+Baidu, ILI%+climate+demography, ILI%+climate+Baidu, ILI%+demography+Baidu, and ILI%+climate+demography+Baidu) to compare the effects of the different combinations. The explained variance score of ILI%+climate+demography+Baidu reached 0.78 and the *R^2^* reached 0.76, which were higher than the corresponding values obtained for the other combinations. Similarly, the mean absolute error of ILI%+climate+demography+Baidu was 0.08 and the mean squared error was 0.01, which were lower than the corresponding values of the other combinations (Table 3). This showed that the ILI%+climate+demography+Baidu model had the best prediction effect. We then drew a prediction diagram for the ILI%+climate+demography+Baidu model. These results also showed that the model can accurately predict the two peaks from the 46th week in 2018 to the 25th week in 2019 (Figure 3).

**Table 3:** Multiattention-long short-term memory (MAL) deep-learning model of influenza-like illness case proportion (ILI%) in various combinations with other source data.

Data source combinations	*R^2^*	Explained variance score	Mean absolute error	Mean squared error
ILI%	0.6365	0.7331	0.1107	0.0228
ILI%+climate	0.6702	0.6996	0.0896	0.0207
ILI%+demography	0.6830	0.7504	0.0955	0.0199
ILI%+Baidu	0.7022	0.7455	0.0936	0.0187
ILI%+climate+demography	0.6952	0.7037	0.0916	0.0191
ILI%+climate+Baidu	0.6998	0.7438	0.0757	0.0188
ILI%+demography+Baidu	0.6801	0.7660	0.0893	0.0201
All^a^	0.7638	0.7801	0.0765	0.0148

^a^All data sources included: ILI%+climate+demography+Baidu.

### MAL of ILI%×Positive% and Other Data Sources

**Figure 4:**
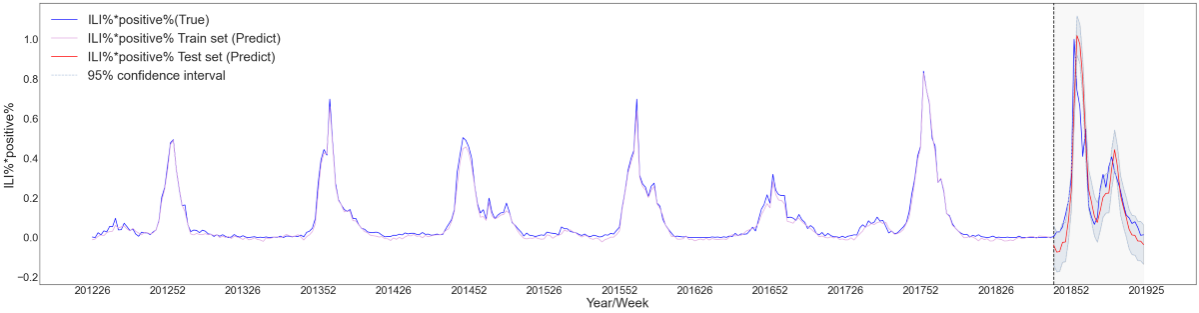
Prediction diagram for ILI%*positive%+climate+demography+Baidu multiattention long short-term memory (MAL) deep-learning model. ILI%*positive%: product of the percentage of influenza-like illness cases and the influenza-positive rate.

We first used the MAL to model ILI%×positive% alone, and then combined and modeled ILI%×positive% with climate, demography, and Baidu data in different forms (ILI%×positive%+climate, ILI%×positive%+demography, ILI%×positive%+Baidu, ILI%×positive%+climate+demography, ILI%×positive%+climate+Baidu, ILI%×positive%+demography+Baidu, and ILI%×positive%+climate+demography+Baidu) to compare the effects of the different combinations. The explained variance score of ILI%×positive%+climate+demography+Baidu reached 0.74 and the *R^2^* reached 0.70, which were higher than the corresponding values of the other combinations. Similarly, the mean absolute error of ILI%×positive%+climate+demography+Baidu was 0.02 and the mean squared error was 0.02, which were lower than the corresponding values of the other combinations (Table 4). This showed that the ILI%×positive%+climate+demography+Baidu model had the best prediction effect. We then drew a prediction diagram for the ILI%×positive%+climate+demography+Baidu model. The results also showed that the model can accurately predict the two peaks from the 46th week in 2018 to the 25th week in 2019 (Figure 4).

**Table 4:** Multiattention-long short-term memory (MAL) deep-learning model of the product of the percentage of influenza-like illness cases and influenza-positive rate (ILI%×positive%) in combination with other source data.

Data source combinations	*R^2^*	Explained variance score	Mean absolute error	Mean squared error
ILI%×positive%	0.5593	0.5719	0.1128	0.0234
ILI%×positive%+climate	0.6609	0.6610	0.0886	0.0180
ILI%×positive%+demography	0.6556	0.6942	0.0950	0.0183
ILI%×positive%+Baidu	0.6375	0.6539	0.0982	0.0192
ILI%×positive%+climate+demography	0.6613	0.6690	0.0911	0.0179
ILI%×positive%+climate+Baidu	0.6449	0.7071	0.0941	0.0188
ILI%×positive%+demography+Baidu	0.6788	0.6829	0.0898	0.0170
All^a^	0.7025	0.7396	0.0158	0.0161

^a^All data sources included: ILI%×positive%+climate+demography+Baidu.

### Comparison of ILI% and Other Source Data Between Different Models

We modeled different combinations of ILI% and other data sources, and compared the performance of traditional machine-learning models (random forest and XGBoost) and other deep-learning models (LSTM and GRU) with that of our MAL model. The results showed that the MAL model with the ILI%+climate+demography+Baidu combination had the best prediction effect. The *R^2^* was 0.76 and the explained variance score was 0.78, which were higher than the corresponding values of the other models; the mean absolute error was 0.08 and the mean squared error was 0.01, which were lower than the corresponding values of the other models (Table 5).

**Table 5:** Comparison of the percentage of influenza-like illness cases (ILI%) and combinations of other source data between different models.

Model performance		ILI%	ILI%+climate	ILI%+demography	ILI%+Baidu	ILI%+climate+demography	ILI%+climate+Baidu	ILI%+demography+Baidu	All
***R^2^***									
	RF^a^	–0.0564	0.0121	0.0657	–0.0219	0.1345	0.1062	0.0835	0.0531
	XGB^b^	0.3841	0.3425	0.3827	0.3197	0.3743	0.3276	0.3153	0.3327
	LSTM^c^	0.5891	0.6102	0.5919	0.5621	0.5661	0.5718	0.5705	0.6116
	GRU^d^	0.4775	0.5387	0.5232	0.5263	0.5206	0.5768	0.5781	0.6461
	MAL^e^	0.6365	0.6702	0.6831	0.7022	0.6952	0.6998	0.6801	0.7638
**Explained variance score**									
	RF	0.3349	0.3735	0.4271	0.3178	0.4778	0.4603	0.4524	0.3449
	XGB	0.4567	0.4180	0.4545	0.4162	0.4372	0.4108	0.4157	0.4102
	LSTM	0.7283	0.7353	0.6833	0.6974	0.7336	0.7131	0.6955	0.6901
	GRU	0.6027	0.6195	0.6229	0.6876	0.6292	0.6686	0.6821	0.7006
	MAL	0.7331	0.6996	0.7504	0.7455	0.7037	0.7438	0.7660	0.7801
**Mean absolute error**									
	RF	6.4062	6.2187	6.2188	6.4687	6.0625	6.2187	6.3437	6.4062
	XGB	4.8522	4.9781	4.8980	5.0473	4.9431	4.9963	5.1084	4.9209
	LSTM	0.1156	0.1062	0.1048	0.1093	0.1138	0.1066	0.1043	0.0947
	GRU	0.1205	0.1101	0.1131	0.1171	0.1068	0.1034	0.1082	0.1037
	MAL	0.1107	0.0896	0.0955	0.0936	0.0916	0.0757	0.0893	0.0765
**Mean squared error**									
	RF	110.7812	103.5937	97.9687	107.1562	90.7501	93.7187	96.0937	99.2812
	XGB	64.5927	68.9396	64.7296	71.3257	65.6069	70.5054	71.7936	69.9706
	LSTM	0.0258	0.0244	0.0256	0.0275	0.0272	0.0268	0.0269	0.0243
	GRU	0.0328	0.0289	0.0299	0.0297	0.0301	0.0265	0.0265	0.0222
	MAL	0.0228	0.0207	0.0199	0.0187	0.0191	0.0188	0.0201	0.0148

^a^RF: random forest.

^b^XGB: extreme gradient boosting.

^c^LSTM: long short-term memory.

^d^GRU: gated current unit.

^e^MAL: multiattention-long short-term memory.

### Comparison of ILI%×Positive% and Other Source Data Between Different Models

We modeled different combinations of ILI%×positive% and other data sources, and compared the performance of traditional machine-learning models (random forest and XGBoost) and other deep-learning models (LSTM and GRU) with that of our MAL model. The results showed that the MAL with the ILI%×positive%+climate+demography+Baidu combination had the best prediction effect. The *R^2^* was 0.70 and the explained variance score was 0.74, which were higher than the values of the other models; the mean absolute error was 0.02 and the mean squared error was 0.02, which were lower than the values of the other models (Table 6).

**Table 6:** Comparison of the product of the proportion of influenza-like illness cases and influenza-positive rate (ILI%×positive%) in combination with other data sources between different models.

Model performance		ILI%×positive%	ILI%×positive%+climate	ILI%×positive%+demography	ILI%×positive%+Baidu	ILI%×positive%+climate+demography	ILI%×positive%+climate+Baidu	ILI%×positive%+demography+Baidu	All
***R^2^***									
	RF^a^	0.0174	0.0351	–0.0975	0.0548	–0.0662	0.1066	0.0563	0.2482
	XGB^b^	0.4559	0.4674	0.4843	0.6299	0.4578	0.5856	0.6131	0.6241
	LSTM^c^	0.4796	0.4933	0.5037	0.4781	0.5966	0.5976	0.6026	0.6321
	GRU^d^	0.4402	0.5178	0.5082	0.4817	0.5401	0.5396	0.5226	0.6322
	MAL^e^	0.5593	0.6609	0.6556	0.6375	0.6613	0.6449	0.6788	0.7025
**Explained variance score**									
	RF	0.3268	0.3721	0.2985	0.3273	0.2879	0.4213	0.3711	0.3615
	XGB	0.4617	0.4678	0.4886	0.6357	0.4617	0.5859	0.6259	0.6305
	LSTM	0.4996	0.4938	0.6501	0.6225	0.6801	0.6568	0.6038	0.6679
	GRU	0.5665	0.6304	0.6191	0.5904	0.5607	0.6162	0.6046	0.6381
	MAL	0.5719	0.6610	0.6942	0.6539	0.6690	0.7071	0.6829	0.7396
**Mean absolute error**									
	RF	4.0625	4.1562	4.4687	4.2187	4.2500	3.9687	4.0312	3.4687
	XGB	2.8446	3.1022	2.8304	2.5067	3.0427	2.7829	2.5261	2.5808
	LSTM	0.1146	0.1107	0.1172	0.1221	0.0951	0.1045	0.1022	0.0868
	GRU	0.1134	0.1001	0.1015	0.1164	0.1081	0.1185	0.1097	0.0877
	MAL	0.1128	0.0886	0.0950	0.0982	0.0911	0.0941	0.0898	0.0158
**Mean squared error**									
	RF	40.3125	39.5937	45.0312	38.7812	43.7500	36.6562	38.7187	30.8437
	XGB	22.3243	21.8500	21.1579	15.1839	22.2425	16.9991	15.8741	15.4241
	LSTM	0.0275	0.0268	0.0263	0.0276	0.0213	0.0213	0.0211	0.0195
	GRU	0.0296	0.0255	0.0260	0.0274	0.0243	0.0244	0.0253	0.0194
	MAL	0.0234	0.0180	0.0183	0.0192	0.0179	0.0188	0.0170	0.0161

^a^RF: random forest.

^b^XGB: extreme gradient boosting.

^c^LSTM: long short-term memory.

^d^GRU: gated current unit.

^e^MAL: multiattention-long short-term memory.

## Discussion

### Principal Findings

Our research purpose was to explore a new, better-performing deep learning model to predict influenza trends from multisource heterogeneous data in a megacity. The highest correlation coefficients were found for Baidu query data for ILI% and air quality for ILI%×positive%. In addition to these variables, temperature, sunlight, air pressure, humidity, and wind speed had high correlation coefficients for ILI%, whereas temperature, Baidu query data, air pressure, wind speed, and humidity had high correlations for ILI%×positive%. We established the MAL model and predicted the ILI% and ILI%×positive%. We also combined the data in different forms and evaluated the performance of several common machine-learning and deep-learning models to predict influenza trends for comparison. We first used the MAL to model ILI% and then combined and modeled ILI% with climate, demography, and Baidu data in different forms. We found that the ILI%+climate+demography+Baidu combination had the best prediction effect. Similarly, we used the MAL to model ILI%×positive% and combined this with different data forms. We found that the ILI%×positive%+climate+demography+Baidu combination had the best prediction effect. The comparison with random forest, XGBoost, LSTM, and GRU models showed that the newly established MAL model had the best prediction effect using multisource data.

The combination of multisource heterogeneous data and multichannel surveillance could reduce forecasting errors introduced from a prediction based purely on internet-derived data or climatic data. The fact that the models combining ILI% or ILI%×positive% with climate, demography, and Baidu search data had the best prediction effect indicated that the emergent risk of seasonal influenza could be assessed using multiple forms of surveillance data. Both internet-based query and climate data have been previously suggested in developing predictive models for climate-sensitive infectious diseases based on spatiotemporal models [26]; traditional surveillance data are necessary and the moderate symptomatic surveillance system could be further exploited. The WHO has developed “pandemic and epidemic intelligence” as a new model for the surveillance of emerging threats, which is expected to expand for increased anticipation and preparation for future threats. Traditional surveillance is inadequate when only data for confirmed cases are available [27]. Risk factors and the absence of symptomatic data lead to reduced detection of multipoint triggering mechanisms for infectious diseases. Therefore, moderate surveillance channels must be created to increase detection sensitivity and accuracy.

In our results, the search engine data had one of the highest correlation coefficients for ILI% and proved to be a powerful variable for estimating epidemic status. During the current COVID-19 pandemic, Twitter could estimate the prevalence of COVID-19 in the United States [28]. Baidu data could also be used to forecast dengue fever. However, studies have also shown that some search engines may overestimate peak amplitude [29]. Therefore, investigating the regulations of various combined keywords and search policies in search engine data by using machine-learning methods will be necessary in future studies. Based on the findings of this study, we also encourage the use of search engine query surveillance, particularly in developing countries and regions with the greatest number of internet users, such as Asia (53.6%) and Africa (11.9%) [30].

Natural factors may substantially influence the epidemic of respiratory infections more than social factors. Our study showed that natural factors had a greater impact weight on ILI cases, whereas economic factors such as GDP, holidays, and social factors had a lower impact weight on ILI cases. Several studies have indicated that climate is the key factor in forecasting climate-sensitive infectious diseases such as dengue and other vector-borne diseases. Seasonal influenza is also a climate-sensitive disease. However, social measures have remained stable. Therefore, we have not found a significant impact of social factors, because the COVID-19 pandemic response provided an opportunity to identify the social public health measures that have a significant impact on influenza protection [31-33].

Deep learning is a new aspect of machine learning. In recent years, deep learning has made unprecedented achievements in classification, detection, recognition, prediction, and other tasks, and has attracted extensive attention from all levels of society. Deep learning can solve problems that are difficult to solve by traditional machine learning, such as high-dimensional and jumbled data. Owing to the COVID-19 epidemic, there has been a gradual increase in exploration and research on the prediction and early warning of respiratory infectious diseases using deep learning. Yi et al [34] improved pneumonia surveillance ability using a revolutionary, scalable, and interpretable deep neural network. Jung et al [35] used a deep-learning model based on self-attention to predict influenza in a region, and the results showed that the model was effective in predicting the trend of influenza. An overview study summarized the application of existing deep-learning and medical image analysis methods, systematically discussed the problems associated with deep-learning methods and the COVID-19 imaging mode, and reported several promising research results [36].

In the past, the influenza surveillance of the CDC in China was mainly conducted using only confirmed case data; hence, there was a time lag in influenza surveillance. In the future, we should prepare for respiratory infectious disease epidemics, especially emerging acute respiratory infectious diseases. Therefore, it is important to improve and optimize the prediction and early warning system of respiratory infectious diseases. Researchers began to use machine learning, deep learning, artificial intelligence, and other methods in combination with big data to establish multichannel and multisource prediction and early warning models. Furthermore, it is essential to establish a trinity working system of detection, decision-making, and early warning. In this study, we first used ILI cases, search engine, and other data, which occurred before the case was confirmed, to predict the outbreak of influenza in advance. In addition, owing to the diversity of data sources, it is difficult for traditional models to simultaneously input multiple variables for prediction. Therefore, we innovatively established a deep-learning model (MAL), which can model ILI cases and other monitoring data from different sources at the same time. The results also showed that this model has a good effect on predicting the trend of seasonal influenza. Therefore, we believe that this study can advance the time of influenza outbreak prediction and provide a reference for future technologies to predict influenza trends from different sources. Thus, our research can help establish an auxiliary decision-making system for public health emergencies.

### Study Limitations

This study had a few limitations. First, owing to the limited availability of data, many influencing factors and symptom surveillance data were not available or sufficiently detailed. Second, circulating virus strains, specimen collection rates, case selection bias, and health care–seeking behaviors could affect virological surveillance. Finally, we innovatively used a deep-learning model to predict the trend of influenza in a megacity. Although this method is applicable to seasonal influenza, it may not be applicable to the prediction of chronic respiratory infectious diseases.

### Conclusions

In summary, our findings demonstrated that the MAL model performs better in predicting influenza than traditional machine-learning and deep-learning models. The prediction and early warning model for influenza and other acute respiratory infectious diseases still needs to be improved. We believe that the model developed in this study can advance the time of influenza outbreak prediction and provide a reference for future technologies to predict influenza trends from different sources. Based on further timely and effective prediction of influenza epidemic intensity, early and better preparedness can be implemented to reduce the health damage to the population.

## Abbreviations

AH: absolute humidity
Apmean: mean air pressure per week
AQI: air quality index
CDC: Center for Disease Control and Prevention
GDP: gross domestic product
GRU: gated recurrent units
ILI: influenza-like illness
LSTM: long short-term memory
MAL: multiattention-long short-term memory
RH: relative humidity
Tmax: maximum temperature per week
Tmean: mean temperature per week
Tmin: minimum temperature per week
WHO: World Health Organization
XGBoost: extreme gradient boosting

## Data Availability

Climate data were obtained from China Meteorological Data Sharing Service System [20]. The air quality data were from Beijing Municipal Ecological and Environmental Monitoring Center [23]. The search index came from the Baidu search index engine [25]. GDP data were from Beijing Municipal Bureau of Statistics [24]. The data of influenza-like illness cases and influenza etiology in this study were from Beijing Center for Disease Prevention and Control and the Chinese Center for Disease Control and Prevention. The data sets analyzed during the current study are available from the corresponding author on reasonable request.
